# Spatiotemporal distribution of different extracellular polymeric substances and filamentation mediate *Xylella fastidiosa* adhesion and biofilm formation

**DOI:** 10.1038/srep09856

**Published:** 2015-04-20

**Authors:** Richard Janissen, Duber M. Murillo, Barbara Niza, Prasana K. Sahoo, Marcelo M. Nobrega, Carlos L. Cesar, Marcia L. A. Temperini, Hernandes F. Carvalho, Alessandra A. de Souza, Monica A. Cotta

**Affiliations:** 1 Applied Physics Department, Institute of Physics ‘Gleb Wataghin’, State University of Campinas, 13083-859, Campinas, São Paulo, Brazil; 2 Citrus Center APTA ‘Sylvio Moreira’, Agronomic Institute of Campinas, 13490-970, Cordeirópolis, São Paulo, Brazil; 3 Fundamental Chemistry Department, Institute of Chemistry, University of São Paulo, 05508-000, São Paulo, Brazil; 4 Quantum Electronics Department, Institute of Physics ‘Gleb Wataghin’, State University of Campinas, 13083-859, Campinas, São Paulo, Brazil; 5 Structural and Functional Biology Department, Institute of Biology, State University of Campinas, 13083-865, Campinas, São Paulo, Brazil

## Abstract

Microorganism pathogenicity strongly relies on the generation of multicellular assemblies, called biofilms. Understanding their organization can unveil vulnerabilities leading to potential treatments; spatially and temporally-resolved comprehensive experimental characterization can provide new details of biofilm formation, and possibly new targets for disease control. Here, biofilm formation of economically important phytopathogen *Xylella fastidiosa* was analyzed at single-cell resolution using nanometer-resolution spectro-microscopy techniques, addressing the role of different types of extracellular polymeric substances (EPS) at each stage of the entire bacterial life cycle. Single cell adhesion is caused by unspecific electrostatic interactions through proteins at the cell polar region, where EPS accumulation is required for more firmly-attached, irreversibly adhered cells. Subsequently, bacteria form clusters, which are embedded in secreted loosely-bound EPS, and bridged by up to ten-fold elongated cells that form the biofilm framework. During biofilm maturation, soluble EPS forms a filamentous matrix that facilitates cell adhesion and provides mechanical support, while the biofilm keeps anchored by few cells. This floating architecture maximizes nutrient distribution while allowing detachment upon larger shear stresses; it thus complies with biological requirements of the bacteria life cycle. Using new approaches, our findings provide insights regarding different aspects of the adhesion process of *X. fastidiosa* and biofilm formation.

A crucial aspect of pathogenicity of some bacteria is the ability to form a collective body (biofilm) in the host. Basically, a biofilm is a community of microorganisms attached to a surface and embedded in a self-produced matrix of hydrated extracellular polymeric substances (EPS)^1^. The currently accepted model^2^ assumes five stages for biofilm formation: a) reversible adhesion of planktonic cells; b) irreversible adhesion; c) EPS matrix formation; d) biofilm maturation; and e) dispersion. In biofilm literature, it is common ground to compare samples from wild-type and/or mutant cells, cultivated in different conditions, against each other. The observed differences are usually attributed to the altered environmental condition. However, sometimes only the biofilm evolution rate is altered, and not its intrinsic features or evolutionary stages. In fact, bacterial growth usually exhibits statistical similarities^3^,^4^; it is thus important to create a reference framework for biofilm growth dynamics, against which to compare morphological features of samples cultivated under different conditions, even with the same growth time. To fulfill this goal, several aspects of the stages proposed in the current model^2^ have yet to be observed in detail during biofilm formation, at single cell or even higher resolutions. Such approach can address many unresolved questions; among them, lies the role of EPS. In general, EPS describes the collection of polysaccharides, DNA oligomers, proteins and peptides^1^ forming the enveloping matrix that shields the pathogen from host defenses^5^. Also, EPS mediates the transition from reversible to irreversible adhesion^6^ of single cells and facilitate further adhesion of motile bacteria^7^,^8^,^9^,^10^. EPS trails, for example, have recently been suggested to facilitate adhesion in the case of bacterial cells exhibiting surface motility^7^,^8^,^9^. Furthermore, the early spatial organization of this extracellular matrix in the case of living *Vibrio cholerae* biofilms^10^ has shown the complementary architectural roles of matrix proteins and extracellular polysaccharides to eventually form a mature biofilm. On the larger scale of bacterial life cycle, Ma *et* *al*.^11^ have evaluated the role of Psl polysaccharide in *Pseudomonas aeruginosa* biofilms. Psl is associated to surface cells in a helicoidal pattern at early developmental stages, allowing cell-cell adhesion and multiple layers of cell aggregates. Later on, Psl serves as a fibrous, matrix substance to enmesh the bacteria in biofilm. The authors, however, acknowledge that their data may not be extrapolated to other systems.^11^ In fact, most studies show limited snapshots of isolated stages of biofilm development for different bacteria or focus on the role of a specific actor throughout the process; notwithstanding, a solid understanding on biofilm formation should benefit from consistent observations along the whole biological cycle of a single bacterial species.

With this in mind, we have performed a single cell resolution study of the biofilm formation process of Gram-negative *Xylella fastidiosa* bacteria, a generalist phytopathogen which shares common genetic traits with human bacterial pathogens^12^,^13^,^14^. *Xylella fastidiosa* causes diseases worldwide in a wide range of important crops such as citrus, grape, coffee, almond, olives, among others^15^,^16^,^17^. Originally inhabiting parts of the American continent, *X. fastidiosa* has conquered new geographical regions in several other continents as well as new plant host species^16^,^17^,^18^. *X. fastidiosa* thus causes severe economic losses and has recently been listed as one of the top-ten most studied phytopathogenic bacteria^19^. During its life cycle, this microorganism forms biofilm in the foregut of xylem-feeding sharpshooters leafhoppers (*Cicadellidae*) and spittlebugs (*Cercopidae*) vectors^20^. In plant, the bacterial cells attach to the xylem and multiply, forming biofilms^21^; at high cell densities a cell-cell signaling mechanism is required for the bacterium acquisition by the insect vector and, therefore, the subsequent transmission to healthy plants^22^. Thus, biofilm formation is a key process for *X. fastidosa* lifestyle, both in plant and insect vector^21^,^22^, being essential for bacteria spreading and survival. It has been shown that the ability of the bacteria to move and become systemic within the xylem vessels is essential for the development of symptoms in the plant^22^,^23^; biofilm formation seems to attenuate the pathogen’s virulence by enhancing cells attachment to surfaces, inhibiting fast colonization through the vessels and consequently delaying symptoms expression^22^. However, when sufficiently large, biofilms occlude the xylem vessels, disturbing water and nutrient transport^21^,^24^,^25^,^26^.

Many studies have so far contributed to the genetic understanding of *X. fastidiosa* biofilms. It has been shown that type I and type VI pili, afimbrial proteins as well as mineral elements play key roles in the initial cell-surface attachment, cell-cell aggregation process and biofilm development^12^,^27^,^28^,^29^,^30^,^31^,^32^. In addition, the production and secretion of EPS seems also to influence initial adhesion^33^, biofilm formation, plant virulence, and insect transmission^34^. In order to integrate the knowledge about *X. fastidiosa* biofilms into a consistent model, which unveil vulnerabilities leading to control strategies of infected plants, the detailed understanding of adhesion and biofilm formation mechanisms is crucial.

To create an experimentally validated scenario from the initial adhesion of planktonic cells to changes in phenotypes and biofilm structuring at single cell resolution, we have used an extensive pool of microscopic and spectroscopic techniques to analyze – with resolution down to the nanometer scale – the culture of cells from both wild-type 9a5c and soluble green fluorescent protein (GFP) expressing 11399 strains of *X. fastidiosa*. Our study unveils novel and unexpected results, which improve the understanding of adhesion and biofilm formation. We have identified and characterized the spatial and temporal distribution of different EPS constituents (soluble S-EPS; tightly bound, TB-EPS; loosely bound, LB-EPS)^35^ during cell adhesion stages and along biofilm maturation as critical steps for the formation of the multicellular assembly. We have also identified the important role of filamentous cells, which interconnect neighboring clusters. The biofilm reaches eventually a floating, weakly anchored architecture, which complies with the biological requirements of *X. fastidiosa* lifestyle^21^.

## Results

### Planktonic cell adhesion and EPS-mediated surface attachment

**Figure 1:**
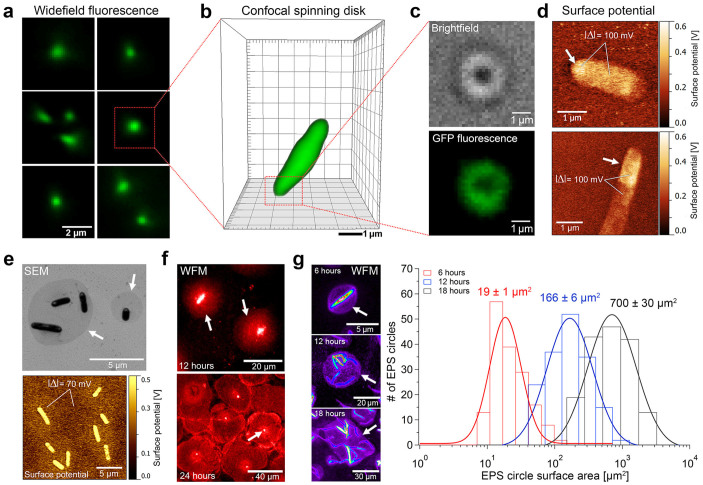
Single cell surface adhesion and irreversible attachment on continuously growing S-EPS areas. (a) *Ex-vivo* WFM images of individual surface-adhered *Xylella fastidiosa* by their polar region. (b) *Ex-vivo* SDCLM images of reversibly adhered bacteria via the polar region. (c) Brightfield and corresponding WFM images reveal circular S-EPS structures at bacterial adhesion regions. (d) SPM images show differences in surface potential (Δ = ~100 mV) indicating S-EPS coating at the polar regions of the bacteria. (e) Contrast difference in SEM image and changes in surface potential (Δ = ~70 mV) identify S-EPS disks around irreversibly attached bacteria. (f) Fluorescence staining (PAS, periodic-acid-Schiff) of polysaccharides show growing circular S-EPS shapes over time. (g) Histogram of S-EPS disks covered area (*n* = *168* for each growth time) demonstrates disk growth with increasing bacteria incubation time (example false-color fluorescence images on left panel for the different growth times of 6, 12 and 18 h). See also Supplementary Figs. S1 and S2. Measurement statistics are described in the Materials and Methods section.

We have first focused our attention to the very first stage of biofilm formation and analyzed the surface adhesion of individual planktonic cells via *ex-vivo* widefield epifluorescence microscopy (WFM) and spinning disk confocal laser microscopy (SDCLM). The obtained WFM data (Fig. 1a) indicate that single bacteria are adhered vertically through the cell polar region (CPR) to the surface; cell precession movement around a vertical axis passing through the pole attached to the surface can be observed (Supplementary Movie S1). Complementary SDCLM data of such individual bacterial cells (Fig. 1b) confirms that the bacteria-substrate adhesion takes place through the CPR. Our Scanning Electron Microscopy (SEM) data of adhered cells (Supplementary Fig. S1) suggest that the initial, reversible cell adhesion could be mediated by fimbrial pili structures, which are mainly located at the CPR, in agreement with previous studies^27^,^28^,^29^. After gentle washing of the sample, the subsequent application of a covalent bacterial structure fixation method^36^ reveals small ring-like S-EPS structures (Fig. 1c, outer and inner diam. 2170 ± 250 nm and 640 ± 90 nm, respectively; *n* = *29*) at the regions where cells were previously reversibly adhered. The central hole diameter of those structures agrees with the observed (Fig. 1b) and expected size for *X. fastidiosa* CPR (varying between ~0.5–1 µm). The observable fluorescence signal is hereby related with the secretion of GFP molecules to the extracellular region and their chemical incorporation within the S-EPS components.

Upon further growth time, bacteria are more firmly-adhered to the surface and remain laterally attached after washing. At this stage, the CPR can be unambiguously differentiated from the body of the bacterium in Scanning Probe Microscopy (SPM) data of dry samples, allowing the interpretation of contrast variations in surface potential, topographic and phase SPM data (Fig. 1d, Supplementary Fig. S1). In particular, when mapping the surface electrical potential (SP-SPM, Fig. 1d), the CPR region demonstrates a significantly larger potential value (corresponding to Δ = ~100 mV, Fig. 1d) in comparison to the bacterial cell body, indicating charge accumulation. We assume that this difference is caused by the S-EPS^35^,^36^,^37^,^38^,^39^,^40^,^41^ deposited on the CPRs. At this same stage of initial biofilm formation, large, disk-shaped, contrast regions are observed by SEM and SP-SPM (Fig. 1e, Supplementary Fig. S2) around these firmly-attached wild-type cells. The SP-SPM data demonstrate an increase of surface potential in such covered regions (Δ = ~70 mV), of similar magnitude to that observed at the bacterial CPRs (Fig. 1d).

Complementary samples (grown for 12 h and 24 h incubation times) were fluorescently stained with periodic-acid-Schiff to visualize charged polysaccharides. The corresponding WFM images (Fig. 1f) show similar disk-shaped surface regions growing over time and thus confirm the presence of polysaccharide components within the deposited S-EPS material^35^,^36^,^37^,^38^,^39^,^40^,^41^. These images show furthermore a brighter spot close to the center of some S-EPS disks (Fig. 1f); we interpret this spot as the “cap” at the cell attachment point.

The disk-shaped S-EPS regions can also be detected by WFM after chemical fixation^36^ of a GFP-expressing bacterial sample without any staining procedure (Fig. 1g). These regions thus represent S-EPS produced and secreted by bacteria since initial surface attachment. The WFM data (Fig. 1g) illustrate isolated cells at the S-EPS disk boundaries, demonstrating larger adhesion on these islands than on the substrate. The disk-covered surface area with S-EPS increases almost exponentially with time (Fig. 1g, histogram and Supplementary Fig. S2) until individual disks eventually merge. The shapes of observed S-EPS regions are thus firstly annular – associated with cells removed by washing – and grow to disk-like structures as the adhesion is further enhanced (Figs. 1c and e–g, respectively).

### Cell-cell adhesion, bacterial aggregation and growth of enveloping LB-EPS

**Figure 2:**
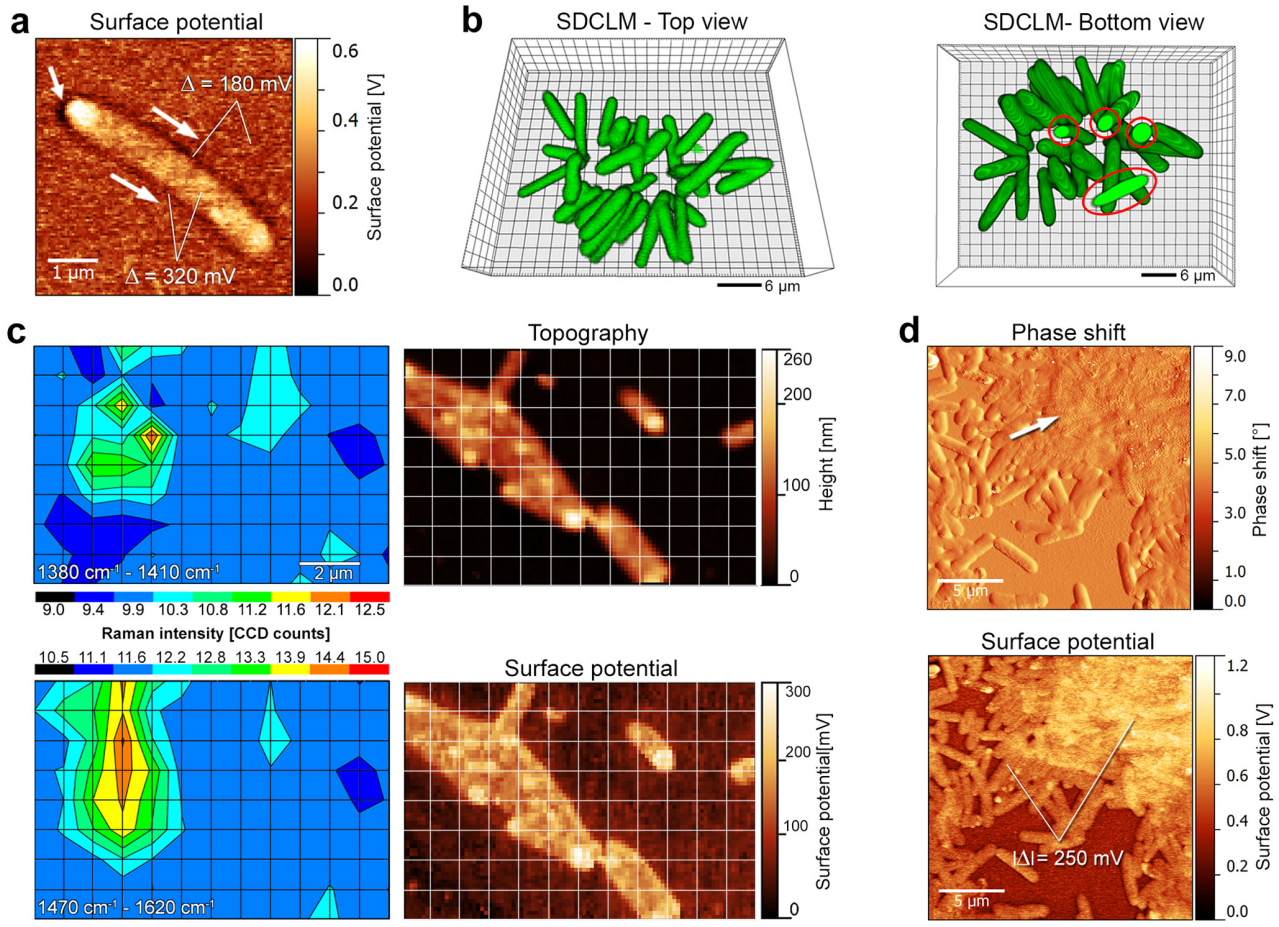
Aggregation of bacterial cells and formation of capsular TB-EPS and cell-cluster enveloping LB-EPS. (a) Capsular, TB-EPS shape observable by surface potential changes in close proximity of the bacterial membrane. (b) *Ex-vivo* SDCLM data of a small bacterial cluster mediated by cell-cell adhesion. The bottom view of the bacterial aggregate demonstrates a small amount of surface-adhered cells (red circles) as bacterial cluster anchorage**.** (c) Confocal Raman intensity data and complementary SPM data of the same sample reveal the presence of polysaccharides (Raman interval 1380 cm^−1^–1410 cm^−1^) and EPS matrix compounds (Raman interval 1470 cm^−1^–1620 cm^−1^). (d) The formation of secondary LB-EPS covering a bacterial cluster can be identified via SPM by significant changes in phase shift (related to material viscoelasticity) and surface potential. See also Supplementary Fig. S3. Measurement statistics are described in the Materials and Methods section.

During further growth of *X. fastidiosa*, SP-SPM images (Fig. 2a) of isolated bacteria show a surface potential drop of Δ = ~180 mV (compared to the substrate) as a dark halo around the entire cell body (Δ = ~320 mV between cell body and halo), suggesting that the CPR “cap” precedes the formation of narrow capsular, tightly-bound TB-EPS^35^. The detectable contrast in surface potential at the cell-substrate interface indicates that S-EPS (substrate) and TB-EPS (cell) exhibit different charge densities according to their chemical compositions. During this biofilm formation stage, planktonic bacteria start to adhere to the more firmly surface-attached cells (Supplementary Fig. S3a, b) and form cell clusters (Fig. 2b, Supplementary Movie S2) with cell-cell junctions via the bacterial CPRs. Interestingly, only the few original cells (Fig. 2b, bottom view, indicated by red circles), are involved in holding these clusters attached to the surface.

The chemical composition of different EPS at diverse biofilm formation stages has been monitored using confocal Raman microscopy (CRM) at selected Raman bands (Fig. 2c). Chemical groups present in carbohydrates^37^ can be detected using a 1380–1410 cm^−1^ band (RB1). This Raman contribution is tentatively attributed to N-Acetylglucosamine (NAG)^38^. Complementary wheat germ agglutinin (WGA) staining experiments to specifically label NAG^39^ on firmly-attached bacteria over a growth time of 24 h (Supplementary Fig. s4a), confirms the assumption that NAG is present in the bacterial surrounding S-EPS layer. A second selected Raman interval (RB2, 1470–1620 cm^−1^ region) corresponds to DNA^40^ and aromatic amino acids (tyrosine and tryptophan), molecules usually associated with LB-EPS matrix in biofilm literature^41^,^42^. Complementary CLSM-data of periodic-acid-Schiff and DAPI stained small biofilm structures (Supplementary Fig. S3c, d) confirms the presence of DNA and polysaccharide material within the biofilm matrix. The spatial distribution of both Raman bands has been compared with SPM-topography images of the same region (Fig. 2c). The RB1 spatial map shows a slightly circular pattern with a few maxima at the bacterial cluster center, where bacterial CPRs with S-EPS accumulation are located. In contrast, RB2 signal is spread around a much larger, elliptically-shaped area around the bacteria cluster.

These results indicate that secondary LB-EPS^35^ has started to be secreted, developing the incipient biofilm matrix. Indeed, in larger clusters, individual cells can no longer be identified in SPM-topography data (Supplementary Fig. S3e), phase shift images (Fig. 2d*,* indicated by white arrow) and SEM images (Supplementary Fig. S3f). The SP-SPM data (Fig. 2d) confirms the presence of the LB-EPS substance on top of the bacterial clusters by demonstrating a drastic change in surface potential of Δ = ~250 mV.

### Interconnecting bacterial clusters by filamentation and vertical biofilm growth

**Figure 3:**
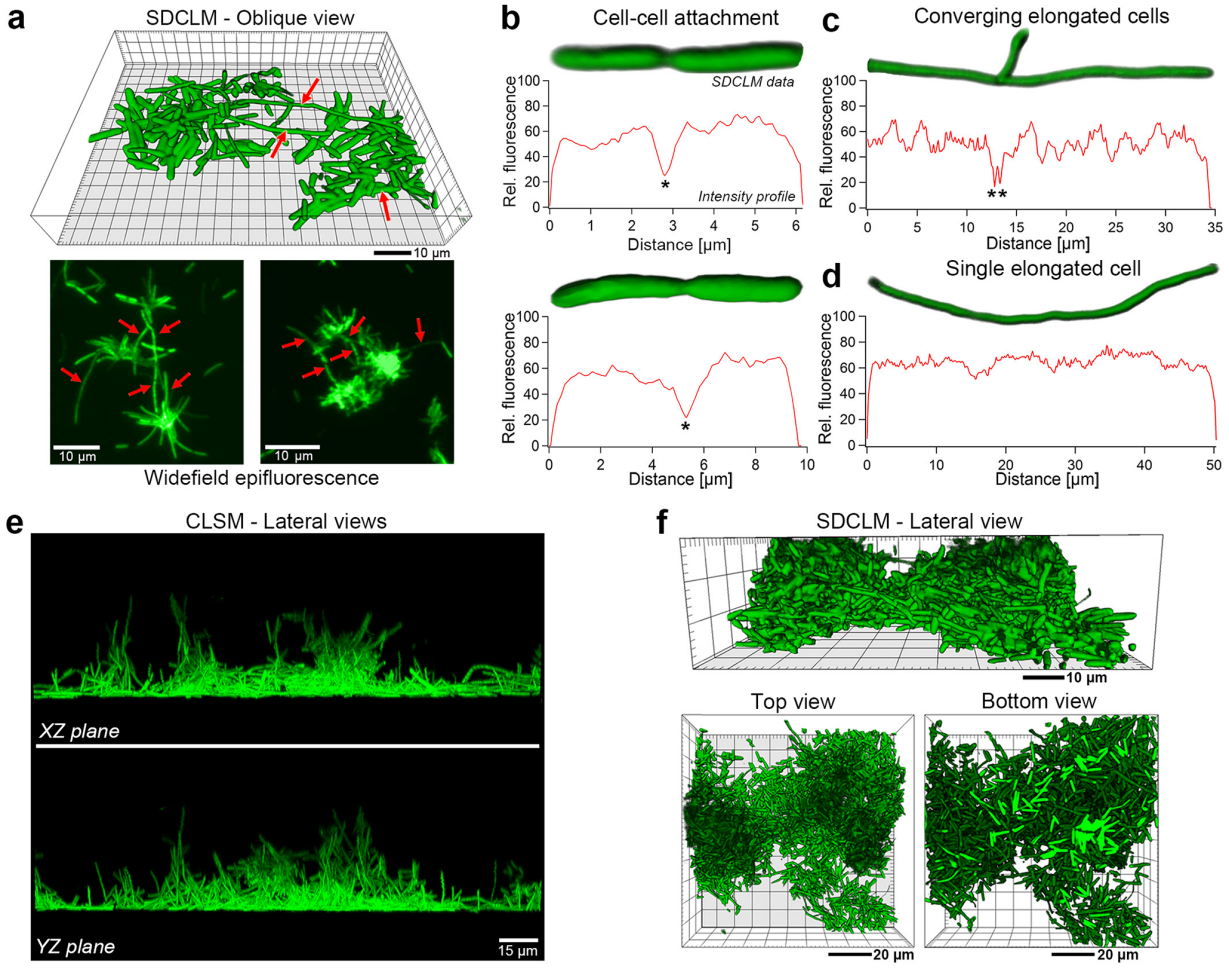
Interconnecting bacterial cluster and vertical biofilm growth. (a) SDCLM and WFM data of small bacterial clusters, interconnected by elongated bacteria (denoted by red arrows). (b–d) *Ex-vivo* SDCLM images and fluorescence intensity profiles of bacterial cell. (b) The gap between two conjoined bacteria is detectable by large drops (denoted with asterisk) in the fluorescence along the bacterial conjunction axis. (c) At the conjunction spot of elongated cells the fluorescence intensity drops similarly, demonstrating cell-cell attachment at these locations. (d) Drastically elongated bacteria that interconnect bacterial cluster demonstrate continuous fluorescence intensity along the bacterial axis. (e) *Ex-vivo* CLSM images show lateral views (XZ and YZ planes) of a bacterial cluster illustrating preferred vertical biofilm growth. (f) *Ex-vivo* SDCLM data of mid-sized, vertical growing interconnected cluster. The bottom view of this small biofilm demonstrates a small number of surface-adhered cells, which can be identified by their light green color. Measurement statistics are described in the Materials and Methods section.

Once matrix formation is initiated, the number of bacterial clusters increases gradually and cluster interconnection is observed in SDCLM and WFM images (Fig. 3a, indicated by red arrows). This process occurs due to an unexpected, dramatic phenotypic change of a few cells located at cluster boundaries. These individual cells elongate up to tens of micrometers to interconnect neighboring clusters as verified by the fluorescence intensity analysis. *Ex-vivo* SDCLM images of conjoined bacterial cells (Fig. 3b, c) show a distinct gap (with a factor 2 reduction, indicated by asterisks) in the fluorescence intensity profiles for cell-cell attached bacteria along the conjunction axis. Contrarily, the elongated cells present a rather continuous profile along the whole cell extension, of up to 50 μm (Fig. 3d), confirming a drastic change in length of individual bacteria. It is important to remind that only GFP expressing bacteria, with large fluorescence signals in the green spectral region, were considered in these experiments, in order to exclude possible contamination as the source of elongated cells.

Upon further biofilm growth, CLSM and SDCLM data of young biofilms demonstrate that biofilm thickening occurs due to the adhesion of planktonic bacteria and division of cells embedded within the biofilm matrix (Fig. 3e). Conversely, the biofilm expansion along the surface occurs at a lower rate (Fig. 3e, f), as vertical growth dominates at surface regions of higher bacterial densities. Despite biofilm expansion, however, the entire structure continues anchored by only few cells from original cluster formations (Fig. 3f, bottom view, Supplementary Movie S3).

### Mature biofilm architecture and growing network of filamentous EPS structures

**Figure 4:**
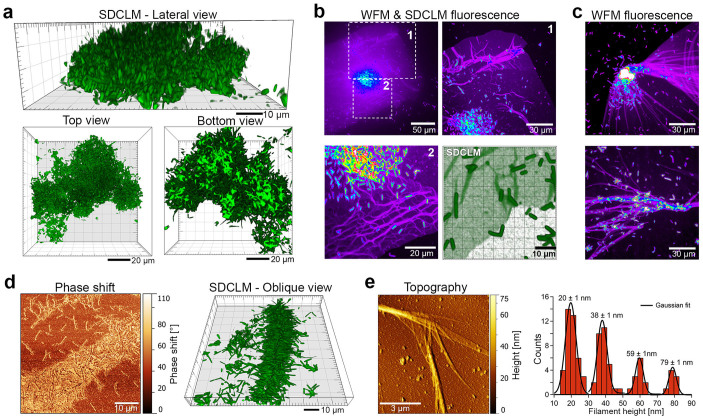
Biofilm architecture and growing network of S-EPS film and filamentous EPS structures. (a) *Ex-vivo* SDCLM data of a mid-sized, vertical growing biofilm. The bottom view of this small biofilm indicates a relatively small number of surface-adhered cells (light green color). (b) False-color WFM and SDCLM fluorescence images identify S-EPS film surrounding a bacterial biofilm and filamentous EPS structures. (c) False-color WFM fluorescence data showing filamentous structures emerging from bacterial biofilm and facilitated bacterial adhesion on the filamentous network. (d) SPM and *ex-vivo* SDLCM data illustrate bacterial adhesion to filamentous EPS structures. (e) AFM topography data of filamentous EPS structures and complementary height distribution of individual EPS filaments (*n* = *89*). See also Supplementary Fig. S4. Measurement statistics are described in the Materials and Methods section.

A thorough scrutiny of the biofilm in maturation (depicted in *ex-vivo* SDCLM images, Fig. 4a) illuminates several aspects of its architecture. After ~72 h of cell growth, mature biofilms continue to exhibit enhanced growth in the vertical direction (Fig. 4a) with thicknesses >40 µm; however, no predominant areas for vertical growth are furthermore observable. Similarly, as observed previously for young biofilms, the entire biofilm structure is anchored to the surface only by a relatively small number of cells, reminiscent of the original irreversibly attached cells (Fig. 4a, bottom view and Supplementary Movie S4).

WFM and SDCLM data of mature biofilm samples (Fig. 4b) show a homogeneous coverage of S-EPS surrounding the biofilms; the boundaries of this S-EPS layer are abrupt and clearly observable. In addition, all fluorescence microscopy datasets (Fig. 4b, c and Supplementary Fig. S4) demonstrate the presence of textured, filamentous structures emerging from biofilms. If no chemical fixation procedure is used, they are easily removed upon gentle washing, thus indicating S-EPS as their main chemical component. The number of individual bacteria over the filamentous region is usually much larger than on the substrate (excluding dense bacterial clusters, see Fig. 4b, c and Supplementary Fig. S4), and similar spatial arrangements are observable with SPM and SDCLM in high cell density regions (Fig. 4d), where the bacterial adhesion is again mediated via their CPRs. Surprisingly, the measured filaments show thickness values which are multiple of ~20 nm, independent from their widths (Fig. 4e).

## Discussion

Our multidisciplinary approach to study individual steps during biofilm formation, allowed us to provide details on the strategies used by the phytopathogenic bacteria *Xylella fastidiosa* in order to create resilient biofilms. Further studies can verify whether these *ex-vivo* strategies also occur under different environments in plant and insect where the pathogen is found^21^.

Regarding our *ex-vivo* results, we show that the initial stage of biofilm formation is represented by the reversible adhesion of planktonic cells to the surface. In agreement to previous works^27^,^28^,^29^, *X. fastidiosa* cells are found to adhere to the surface via (Fig. 1a, b) fimbrial proteins at the CPRs (Supplementary Fig. S1)–mainly type-I and type-IV pili^43^, and also via afimbrial trimeric autotransporters (TAA; adhesins)^13^,^44^. However, these proteins structures do not provide a specific interaction to any specific biomolecule or biotic material (e.g. cellulose or other proteins), as *X. fastidiosa* bacteria is able to adhere to both biotic (e.g. cellulose derivatives) and abiotic (e.g. glass, silicon, gold) substrates, eventually forming biofilms on these surfaces^45^. Previous studies have also shown a direct correlation between divalent cations (Ca^2+^, Mg^2+^) and the adhesion of *X. fastidiosa*^31^,^46^. The TAA structures are highly conserved and structure homology and alignment results determined a coiled ß-roll helical structure for the known afimbrial adhesins (XadA1, XadA2 & XadA3) of *X. fastidiosa*. Yoder *et al*.^47^ suggested that these hollow one-dimensional helical structures can most likely complexate divalent cations, such as Ca^2+^, Mg^2+^, resulting in different net charges of the adhesion proteins. Thus the same protein might respond differently to a surface depending on the availability of divalent cations in the surrounding media or surface. This assumption correlates well with our previous findings of *X. fastidiosa* growth yields correlation to surface potential, and consequently, surface charges^45^. Our data reinforces the assumption that the bacterial adhesion is not only guided by specific molecular recognitions, but seems to be mainly driven by unspecific electrostatic interactions^21^,^48^. This strategy may facilitate surface adhesion under different physico-chemical conditions and thus maximize the efficiency in the complex inter-species (plant-insect) colonization biological life cycle^21^.

Another important result of our observations is that the presence of small amounts of S-EPS around the cells does not necessarily provide irreversible adhesion, using washing as a testing procedure to evaluate adhesion strength. At slightly later stage of biofilm formation, however, our SP-SPM data (Fig. 1d) identifies the secretion and accumulation of S-EPS on the CPRs, producing a distinct electrical signature from the bacteria. At this point, cells remain on the surface after washing, and S-EPS disks are observed around the bacteria, instead of annular shapes with missing cells. By taking as basis previous infrared spectroscopic data^33^, our complementary Raman and WGA-staining experiments analysis (Fig. 2c top panel and Supplementary Fig. S4a) identify this accumulated S-EPS as carbohydrates, mainly N-Acetylglucosamine (NAG)^38^. For the Gram-negative bacteria *Caulobacter crescentus*^49^, NAG is important for the holdfast strength to the surface, while for *X. fastidiosa* this molecule was identified to be crucial for the insect vector colonization^50^. This result is consistent with the cell attachment point also composed for carbohydrates observed by WFM (Fig. 1f). The secretion and further accumulation of S-EPS at the bacterial CPRs (Fig. 1c–f, Supplementary Fig. S1) may thus be considered a valid indicator for the reversible to irreversible cell attachment, representing the second step in the biofilm formation model. The spatial distribution of S-EPS on the substrate around the bacterial cell (Fig. 1e–g, Supplementary Fig. S2) presents rotational symmetry around a vertical axis. Thus we assume the vertically attached cells via CPR as the point source for the continuous deposition of S-EPS. The resulting homogeneous surface coverage by S-EPS renders easier the adhesion of new planktonic/daughter cells (Figs. 1g, 4c, for different growth times); in fact, apparently the S-EPS is continuously secreted during large part of the biofilm life cycle, as discussed further below. Both the substrate coverage by S-EPS and its cell pole accumulation mechanisms present a remarkable difference from the helicoidal pattern of Psl distribution on *Pseudomonas aeruginosa* cells^11^. However, a few Psl regions lying on the surface around single cells can be identified in that same work^11^. Agglomerations of bacterial-secreted materials can also be found in other pathogens; for example, at the initial stage of biofilm formation of the Gram-negative human pathogen *Vibrio cholerae*, the secretion of EPS in combination with matrix proteins mediates their irreversible attachment and is proven to be essential for further biofilm formation^10^. Despite the similar mechanisms, the large disk-shaped regions coated with S-EPS (Fig. 1e–g, Supplementary Fig. S2) seem ubiquitous to *X. fastidiosa*; they could be clearly identified most likely due to the slow bacterial division rate when compared to the rate of S-EPS production.

The third step of biofilm formation is defined by bacterial aggregation and formation of the bacterial enveloping EPS matrix^2^. In the case of *X. fastidiosa*, several parallel processes can be found during this formation step. Once irreversible attachment occurs, bacteria form a narrow EPS layer around the cell body (Fig. 2a), also emerging from the CPRs. This EPS could be identified as tightly-bound TB-EPS^35^, consolidating the holdfast to the S-EPS covered surface. The TB-EPS may serve as irreversible attachment layer for further bacteria attachment, as suggested for Psl in *P. aeruginosa*^11^, as well as an individual protective layer against host defense systems. Hereafter, bacterial cluster formation is driven by cell-cell attachment (Fig. 2b, Supplementary Fig. S3), which is also mediated by specific proteins^12^,^13^,^28^,^30^. The facilitated adhesion of planktonic and daughter cells to the large, S-EPS covered areas, both likewise mediated by the bacterial CPRs, suggests an ubiquitous adhesion mechanism for *X. fastidiosa*.

This process leads to bacterial clusters, which are anchored by few, originally-attached cells. A second layer of EPS starts then to be formed on top of areas with a large number of aggregated cells (Fig. 2d and Supplementary Fig. S3). The analysis of fluorescence microscopy and Raman spectroscopy data (Fig. 2c bottom panel, Supplementary Fig. S3) of this secondary EPS layer identifies a participation of DNA^40^ and aromatic amino acids, such as tyrosine and tryptophan; these organic components can be associated to loosely-bound LB-EPS^35^ according to biofilm literature^41^,^42^. The SPM, SEM and Raman results (Fig. 2d bottom panel, Supplementary Fig. S3 and Fig. 2c, respectively) thus indicate that, at this stage of biofilm formation, LB-EPS started to be secreted, developing the incipient biofilm enveloping matrix, which is intended to shield the biofilm structures from host defense systems^5^. By considering the role of fimbrial and afimbrial proteins, reported in previous works, and the different EPS constituents, shown here, further studies may focus on molecular and biophysical aspects related to the different steps of biofilm formation, which are most likely involved in the lifestyle of this plant pathogen bacterium.

As biofilm formation proceeds, bacterial clusters are formed, and subsequently interconnected by few cells localized at the cluster boundaries. These cells undergo dramatic elongation (Fig. 3a–d), in a phenotypic change that actively establishes bridges between neighboring bacterial aggregations. Bacterial clusters and elongated bacteria have been also reported for other bacterial pathogens. For example, Berk *et* *al.*^10^ have shown that *Vibrio cholerae* initially organizes in clusters; based on results for early adhesion stage, these authors propose that individual clusters expand and passively contact others to form the biofilm^10^. However, elongated cells can be briefly glimpsed, actively bridging clusters in one of their time-lapse microscopy videos^10^, suggesting that the phenotypic change observed here is a more general behavior. Indeed, the phenotypic elongation process, called *filamentation*, has been reported for several bacteria^51^,^52^, including a variety of human-affecting germs. Filamentation is considered as a bacterial adaptation mechanism for survival, often associated with environmental and genetic stresses; the morphological plasticity of pathogenic bacteria could represent a direct and adaptive response to sensing of environmental changes, providing fitter organisms for survival^51^. Among these factors, filamentous cells can also be observed under conditions of high salinity^52^. We can hypothesize a scenario where the spatial proximity of two or more clusters may lead to stronger gradients of continuously secreted molecules used for chemical signaling, in the region between the clusters. Cells located at the cluster borders could sense more easily these changes in the environment, since the cells within the cluster are embedded in the incipient EPS biofilm matrix. The observed filamentation process can thus be a response to the signaling created and regulated by cluster proximity, since our results indicate that *X. fastidiosa* bacteria use filamentation as an active mechanism adapted for biofilm formation. The resulting elongated cell phenotype not only maximizes the probability of bridging clusters but also provides the enhanced coverage of continuously secreted S-EPS of the underlying surface, facilitating cell adhesion. The change in phenotype is thus an essential feature to form an interconnected framework, which represents the rudimentary, basic biofilm structure. To our knowledge, this is the first observation of filamentation for *X. fastidiosa*, a vascular plant pathogen bacterium, and also the first report of filamentous morphologies as a valid mechanism during bacterial biofilm formation.

The experimental observation of biofilm growth and maturation, which represents the fourth and last step of the biofilm formation model, also provide new details, which may point out strategies on plant and insect colonization by *X. fastidiosa*. Due to the facilitated cell-attachment on EPS-covered areas on the framework of interconnected cell clusters, vertical growth dominates, while the expansion along the surface occurs at a significantly lower rate (Figs. 3e, f and 4a). This growth pattern originates the mature biofilm architecture, which is anchored to the surface by only few of the originally-attached cells, reminiscent of the interconnected clusters (Fig. 4a and Supplementary Movie S4). This clustered, floating and therefore weakly anchored structure maximizes nutrient flow at the expense of mechanical stability and adhesion strength (Supplementary Movie S4). This architecture complies with expected requirements for life within both environments (plant or insect) inhabited by the bacteria^21^,^53^. At slow, laminar fluid flow (as in the case of sap, *in planta*), the clustered architecture maximizes nutrient transport; on the other hand, the floating and weakly-anchored architecture is more prone to eventual detachment upon turbulent flow and large shear stresses, which may be expected during insect suction^54^. During biofilm maturation, S-EPS generates a multi-layered filamentous matrix (Fig. 4b–e and Supplementary Fig. S4), which emerges from biofilm structures, facilitates the adhesion of planktonic and daughter cells (Fig. 4c, d) and most likely provides mechanical support. Similar filamentous structures have already been observed as *X. fastidiosa* supporting element within the plant xylem^55^. The observed filamentous architecture may enhance the mechanical stability considering the size/mass limitations associated with bacterial or nutrient diffusion. Filamentous biofilms have been reported for other bacteria^56^ as well, and may provide similar function to the Psl matrix than enmeshes *P. aeruginosa* cells at early stage biofilms^11^. This structural reinforcement might thus represent a more general mechanism. In the particular case of *X. fastidiosa*, the multi-layered structure – as indicated by the multiple heights in the histogram (Fig. 4e) – might be due to the scrolling of continuously secreted S-EPS layers. The scroll structure collapse to the surface, upon removal of culture medium during sample preparation, may lead to the multi-layered and relatively smooth structures observed by SPM. It is important to mention, however, that the filaments should not inhibit cell transmission within hosts due to their observed features, such as solubility and thickness. In conclusion, the observed biofilm architecture complies with expected biological requirements of *X. fastidiosa* inter-species life cycle under different environmental conditions^15^.

Our study thus provides direct observation and experimental validation of important steps of *X. fastidiosa* biofilm formation, starting at single adhesion until biofilm maturation. We identify for the first time different compositions of EPS and their roles during biofilm development, as well as the presence of the elongated cells, resulting from a filamentation process, which could represent an important mechanism associated with environmental adaptation of the bacteria. These new results may be helpful to further improve the current understanding of biofilm formation and also elucidate action mechanisms of recently published potential treatments for infected plants. Recently, Killiny *et al*. pointed out that efforts to disrupt EPS production may result in alternative disease control strategies, since *X. fastidiosa* mutants that do not produce EPS lost the ability to form biofilm, insect transmission, plant movement, and virulence^34^. Indeed, these results concur with the detailed observations reported here, particularly considering S-EPS disks and filaments, which enhance adhesion and add mechanical support to the floating, weakly anchored biofilm. In addition, a recent study provides a possible treatment for *X. fastidiosa* infection, based on the molecule N-acetyl cysteine (NAC), a cysteine analogue used commonly to treat human disease^57^. NAC concentrations over 1 mg/l reduced bacterial adhesion to glass surfaces, biofilm formation and the amount of EPS. No growth was observed for concentrations of 6 mg/ml, much lower than concentrations required for a wide range of bacteria, up to 80 mg/ml^58^. These results reinforce the important roles that EPS plays along *X. fastidiosa* adhesion and life cycle, shown here. The mechanism underlying the interaction of NAC with EPS constituents, however, is still unclear as well as the very moment at which stage during biofilm formation NAC will become effective. With our findings on *X. fastidiosa* biofilm formation and the spatiotemporal distribution of three EPS types, the effect of NAC and other possible biofilm-affecting agents could be studied in more detail, including conditions mimicking more closely the host environment. Moreover, as *X. fastidiosa* shares common genetic traits with other bacteria, our results may thus have much broader outputs.

## Methods

### Bacteria strains

*Xylella fastidiosa* wild type strain 9a5c and the strain 11399^59^, transformed by electroporation with the vector pKLN59^60^ to express soluble GFP (Green Fluorescent Protein), were used in this study. As bacterial growth media, Periwinkle Wilt broth (PW) with Bovine Serum Albumin (BSA) fraction V (Sigma-Aldrich, USA) was used^61^.

### Substrate materials

For Scanning Probe Microscopy (SPM), Scanning Electron Microscopy (SEM) and confocal Raman spectroscopy studies (100) silicon (p-type, doped with boron) substrates (10 cm diameter wafers, Heliodinâmica S.A., Brazil) were used with a gold layer. To obtain such gold-coated surfaces, a 10 nm thick titanium layer was deposited via electron beam physical vapor deposition (ULS600, Oerlikon Balzers, Liechtenstein) at 5 × 10^−7^ Torr to improve the adhesion to the following gold layer. A 150 nm thick Au layer was added afterwards using the same methodological approach. The substrates were cut into rectangular pieces of approximately 1 × 0.5 cm, cleaned with acetone, 2-propanol and deionized water to remove contaminations and sterilized in a final step by oxygen plasma (SE80, Barrel Asher Plasma Technology, USA) for 15 min (50 sccm O_2_, 200 W, 100 mTorr) before bacterial deposition.

For biofilm studies using wide-field epifluorescence microscopy, Confocal Spinning Disk microscopy and Confocal Laser Scanning microscopy, gamma-radiation sterilized 24-well microplates with borosilicate glass bottom (~150 µm thickness) were obtained from Porvair Sciences Ltd., UK. In some cases, square borosilicate cover glasses (type #1, Menzel GmbH, Germany) with a dimension of 22 × 22 mm were used as substrates, also cleaned and sterilized by oxygen plasma as previously described.

### Chemicals

All other buffers and chemicals within this study and mentioned in the text were purchased from Sigma-Aldrich, USA.

### Bacteria plant extraction and pre-inoculum preparation

*Xylella fastidiosa* strains were isolated from petioles of Citrus variegated chlorosis (CVC) symptomatic sweet orange trees hosted in a greenhouse and incubated in PW growth medium. Subsequently, the harvested cells were resuspended in 500 μl of sterile Phosphate-Buffered Saline (PBS). The bacteria concentration via optical density at 600 nm (OD600) was adjusted to OD_600_ = 0.3. Afterwards, the cells were transferred to a 250 ml Erlenmeyer flask containing 50 ml of PW broth and incubated at 28°C in a rotary shaker at 180 rpm for seven days^62^.

### Bacterial growth

Bacterial inoculum with a concentration of 2 × 10^7^ CFU mL^−1^ from the pre-inocula were used for the experiments as initial concentration for biofilm growth studies in PW broth media. The substrates were incubated for different growth times (as mentioned in the respective method descriptions) in a bacterial stove (410/3NDR, Nova ética, Brazil) at 28°C without culture media replacement. After certain growth times, the PW broth media was removed gently and the samples were washed three times with deionized water to remove the constituents of the culture media and non-attached biofilms completely. In a final step, the samples were dried gently in nitrogen flow and temporarily stored at 4–8°C before measurement.

### Covalent EPS fixation

For the wide-field fluorescence microscopy based studies of EPS (Extracellular Polymeric Substances), *Xylella fastidiosa* strains were chemically fixed based on a modified method from Bearinger *et* *al.*^36^ onto borosilicate glass of a 24-well glass bottom microplate. An amount of 500 μl pre-inoculum (2 × 10^7^ CFU mL^−1^) of planktonic bacteria in PW culture media were added to each microplate well and incubated at 28°C for different growth times as stated in the respective manuscript text. After incubation, the culture media was very gently removed and a volume of 500 μl of 10 mM MES (2-(*N*-morpholino)ethanesulfonic acid) buffer (pH 6) containing 50 mM EDC (1-Ethyl-3-(3-dimethylaminopropyl)carbodiimide) was added to the microplate wells. After the chemical coupling reaction at room temperature for 1 h the wells were washed three times with PBS buffer (pH 7.4) for 5 min each. In a final step, the wells were washed shortly with deionized water and dried gently in nitrogen flow.

### EPS filament fixation using glutaraldehyde

For AFM based characterization of filamentous EPS structures, the culture medium was removed gently from inoculated samples after a growth time of 72 h on glass slides. The samples were afterwards incubated for two hours in a PBS (pH 7.4) solution containing 2.5% glutaraldehyde at room temperature. After incubation, the samples were washed gently with PBS buffer for 5 min and twice with deionized water for 30 s each. In a final step, the samples were dried using a weak nitrogen flow.

### Bacterial DNA and polysaccharide labeling

For the identification of neutral polysaccharides, unfixed bacteria samples with a growth time up to 24 h were nitrogen-dried on borosilicate cover glasses and treated with 0.5% periodic acid in deionized water for 10 min before incubation with the Schiff’s reagent for 20 min to obtain a PAS (Periodic Acid-Schiff) staining. After washing thoroughly with deionized water, the bacterial DNA was counterstained with 4 μg ml^−1^ 4′, 6-diamidino-2-phenylindole (DAPI) for 5 min in PBS buffer. A duplicate of PAS and DAPI stained samples were characterized using Confocal Laser Scanning microscopy (CLSM) and wide-field epifluorescence microscopy (WFM).

### Labeling of N-Acetylglucosamine (NAG)

To identify NAG within bacterial secreted S-EPS films, unfixed bacteria samples on borosilicate cover glasses were washed gently with deionized water after a growth time of 24 h and dried gently in nitrogen flow. Afterwards, the samples (*n* = *4*) were passivated with 1% BSA in PBS buffer (pH 7.4) for 5 minutes. After washing gently with PBS buffer, the NAG-staining was perfomed by adding 1 μg/ml Texas Red-labeled wheat germ agglutinin (WGA; Lifetechnologies, USA), followed by incubation for 10 min. After a final washing step with PBS, the samples were measured using wide-field epifluorescence microscopy (WFM).

### Wide-field epifluorescence microscopy

The GFP expressing bacteria samples of different growth times (as indicated in the main text) were measured using an epifluorescence microscope (Nikon TE2000U, USA) with a peltier-cooled back-illuminated EMCCD camera (IXON^3^, 1024 × 1024 pixels, Andor, Ireland) for sensitive fluorescence detection and a 100× oil-immersion objective (CFI APO TIRF, NA. 1.45, Nikon, USA). GFP excitation and bacterial bright-field imaging was achieved by a 150 W Mercury-lamp with filter sets (AHF, Tübingen, Germany) for GFP (488 nm) and neutral density (ND8) filters. For each sample, the bacteria and EPS deposition were measured using brightfield imaging followed by a fluorescence measurement. The reported *ex-vivo* observations were based on analysis of *n* = *125* measurements for 5 repetitions; for dried bacterial samples, we analyzed *n* = *510* image acquisitions over 34 repetitions.

### Spinning disk confocal fluorescence microscopy

*Ex-vivo* bacteria samples of different growth times (as indicated in the main text) were examined in the National Institute of Science and Technology on Photonics Applied to Cell Biology (INFABIC) at the State University of Campinas, using an Axio Observer Z.1 microscope (Carl Zeiss AG, Germany) with an 100× oil-immersion objective (α-Plan-Apochromat, NA. 1.46), an 63× oil-immersion objective (Plan-Apo, NA. 1.4) and an 40× oil-immersion objective (EC-Plan-Neofluar, NA. 1.3). The 3D-images were collected with a confocal scanner (CSU-X1, Yokogawa Electronic Corporation) at 5,000 rpm with a peltier-cooled back-illuminated EMCCD camera (IXON^3^, 512 × 512 pixels, Andor, Ireland) for sensitive GFP fluorescence detection with a 488 nm laser line. The z-stack was performed by using a piezo-actuated z-stage (NanoScanZ, Prior Scientific); the distance between each frame was 200 nm. The 3D-analysis was performed using Imaris software (Bitplane, USA); the reported observations were based on the analysis of *n* = *149* 3D-measurements for 6 repetitions.

### Confocal Laser Scanning Microscopy

Confocal Laser Scanning Microscopy (CLSM) measurements of bacterial samples were performed at the National Institute of Science and Technology on Photonics Applied to Cell Biology (INFABIC), State University of Campinas, using a Zeiss LSM780-NLO confocal microscope on an Axio Observer Z.1 microscope (Carl Zeiss AG, Germany) with an 63× oil-immersion objective (Plan-Apochromat, NA. 1.4). DNA and polysaccharide stained bacteria samples were examined using a laser line of 561 nm for PAS-staining excitation and a 405 nm laser excitation for DNA-staining DAPI. For the bacterial biofilm architecture study the GFP excitation was performed with a 488 nm laser line. Imaging was performed with pinholes set to 1 airy unit for each channel, 1024 × 1024 pixels image format and distances of 370 nm for the z-stack. The 3D-image analysis of the bacterial biofilm architecture was also performed with the Imaris software (Bitplane, USA). The reported observations were based on the analysis of *n* = *47* 3D image acquisitions for 3 repetitions.

### Confocal Raman spectroscopy

Confocal Raman microscopy was used to study the early stages of biofilm formation. Gold-coated silicon substrates were used with bacterial samples of 6 h of growth. Raman excitation was performed with a diode laser at 785 nm and Raman spectra were obtained using a Raman imaging system (inVia, Renishaw, United Kingdom), coupled to a microscope (DM2500M, Leica, Germany) with a low-noise CCD detector (RenCam, Renishaw, United Kingdom) and the use of a 50× objective lens (SM Plan, N.A. 0.55, Olympus, USA). Laser excitation power was kept below 6.5 mW to avoid sample degradation during the measurements. The Raman spectra were acquired using a static grating (1800 L mm^−1^), covering the range between 1380 cm^−1^ and 1715 cm^−1^ (integration time of 10 s, 3 accumulations). A total of *n* = *215* Raman spectra were acquired, from which 117 were considered to create the spatial map shown in Fig. 2c.

### Scanning Probe Microscopy

For the study of the biofilm formation, bacterial samples of different growth times (6 h, 1, 2, 7 and 14 days, in duplicate, and 2 repetitions) were cultured on conductive gold-coated silicon substrates as described before. Topography and phase images were acquired using the non-contact mode of the SPM Agilent 5500 (Agilent Technologies, USA) in combination with the MACIII module for multi-frequency electrical Kelvin-Probe measurements (surface potential images, SP-SPM). The SPM measurements were performed with silicon cantilevers (NSC14, MicroMasch, USA), whereas the SP-SPM studies were carried out using metallized, gold-coated cantilevers (NSC14/Cr-Au, MicroMasch, USA) with a typical resonance frequency of *f_0_* = 160 kHz and a spring constant of *k* = 5 N m^−1^. Scanning speed was typically in the range 1–8 µm s^−1^ according to the image size and an AC electrical bias between 1–3 V was applied for Kelvin-Probe surface potential measurements. In order to prevent oxidation and accumulation of charges on the sample surface, a pure nitrogen atmosphere was applied in the isolated sample chamber during acquisition of the surface potential measurements. SPM images (total number = 798) were acquired in order to characterize the main features of the samples. For SP-SPM in particular, 108 individual bacteria were measured in 27 images for SP values of S-EPS disks (*n* = *18*), S-EPS at CPRs (*n* = *40*) and TB-EPS (*n* = *28*); SP values for LB-EPS were acquired from biofilms (*n* = *5*) in 8 individual images. Filament heights were obtained from *n* = *89* cross sections from 4 images of different localizations, in order to create the histogram in Fig. 4e.

### Scanning Electron Microscopy of adhered bacteria and EPS deposition

The *Xylella* *fastidiosa* bacteria and the soluble EPS deposition were investigated using high-resolution FESEM (Field Emission Scanning Electron Microscopy; FEI Inspect F50). Bacteria cells were incubated with different growth times (6 h, 1, 2, 7 and 14 days) on gold-coated silicon substrates as described before. Before measurement, the samples were washed three times with de-ionized water and afterwards dried gently in nitrogen flow. The samples were analyzed using low electron beam energy (1 keV) and short exposure times during the secondary electron imaging mode. Adopting the Field Emission Gun (FEG) during FESEM measurements provided a high contrast image with low electrostatic distortion resulting in spatial resolution ~2 nm. For analysis, *n* = *107* SEM images were measured over 5 repetitions.

## Author Contributions

R.J. and D.M.M. performed bacterial growth, sample preparations, SPM measurements, fluorescence microscopy experiments and data analysis. B.N. performed bacteria plant extraction and pre-inoculum preparation. M.M.N. collected confocal Raman data and discussed the data obtained. P.K.S. performed Scanning Electron Microscopy studies. H.F.C. suggested and carried out the bacterial DNA and polysaccharide staining. A.A.S. provided the biological material and discussed the results from the microbiological point of view. M.L.A.T. and C.L.C. granted access to the confocal Raman and confocal fluorescence microscopy equipment, respectively, and discussed the data obtained. R.J., D.M.M. and M.A.C. designed the study and wrote the article. R.J. and D.M.M. contributed equally to the study. M.A.C. supervised and managed the whole study. All authors discussed the results, commented and approved the manuscript.

## Supplementary Material

Supplementary Information Supplementary Movie 1

Supplementary Information Supplementary Movie 2

Supplementary Information Supplementary Movie 3

Supplementary Information Supplementary Movie 4

Supplementary Information Supplementary Information
