# Supplementary material for: Spatiotemporal distribution of different extracellular polymeric substances and filamentation mediate Xylella fastidiosa adhesion and biofilm formation
Source: Sci Rep. 2015 Apr 20;5:9856. doi: 10.1038/srep09856 (PMC4402645; doi:10.1038/srep09856)
Supplement: Supplementary Information [file srep09856-s5.pdf]

Spatiotemporal distribution of different extracellular polymeric substances and filamentation mediate *Xylella fastidiosa* adhesion and biofilm formation

Richard Janissen, Duber M. Murillo, Barbara Niza, Prasana K. Sahoo, Marcelo M. Nobrega, Carlos L.Cesar, Marcia L. A. Temperini, Hernandes F. Carvalho, Alessandra A. de Souza, Monica A. Cotta

Supplementary Information

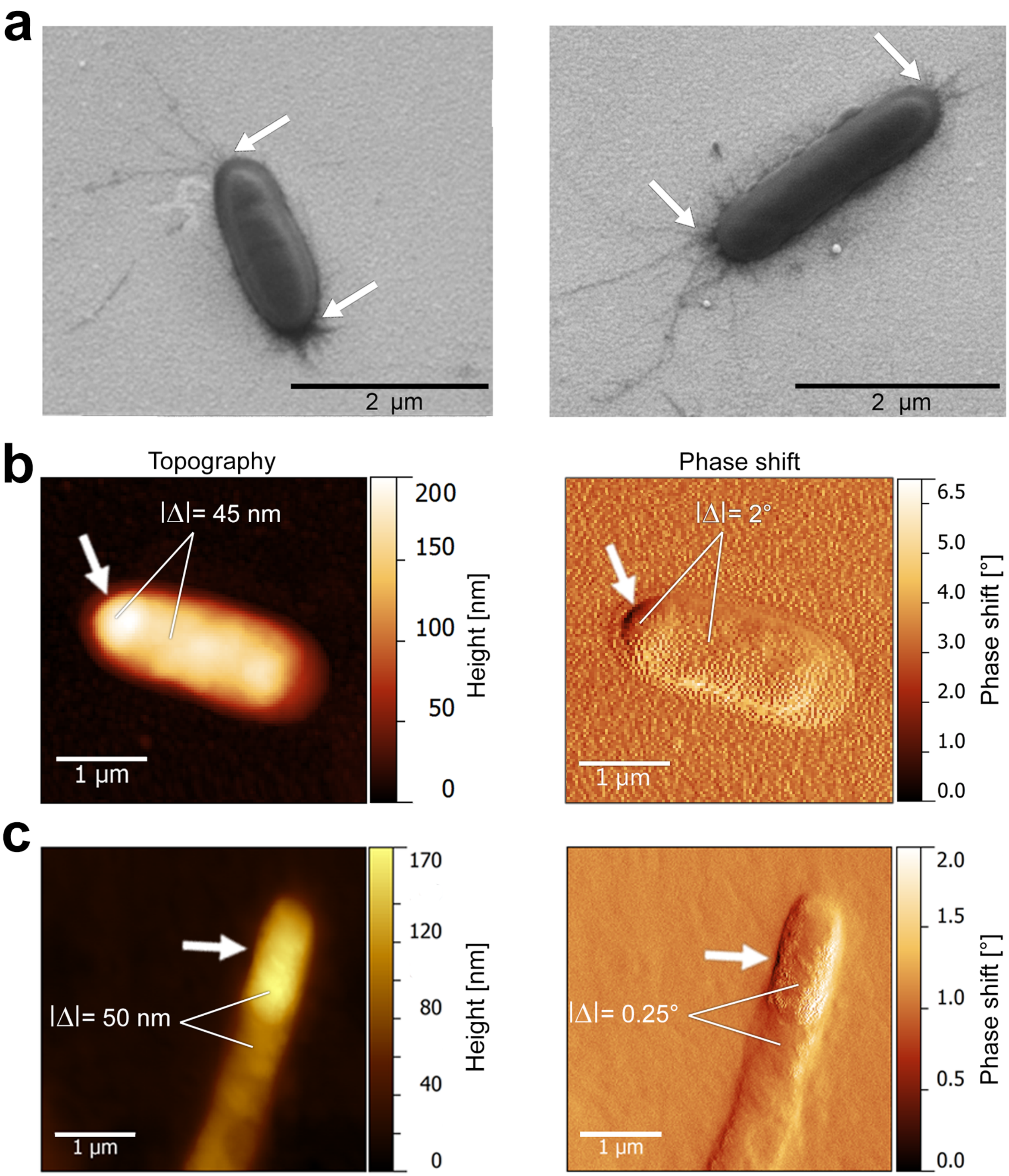

Supplementary Figure S1 | Bacterial pili and S-EPS caps at bacterial CPR, related to Figure 1. (**a**) SEM micrographs of surface-adhered bacteria show the existence of pili emerging from the CPR (indicated by white arrows). (**b**, **c**) SPM topography and phase data illustrate significant changes in height and phase shift at the CPR of individual bacterial cells, indicating the formation of S-EPS at the bacterial polar regions (denoted by white arrows).

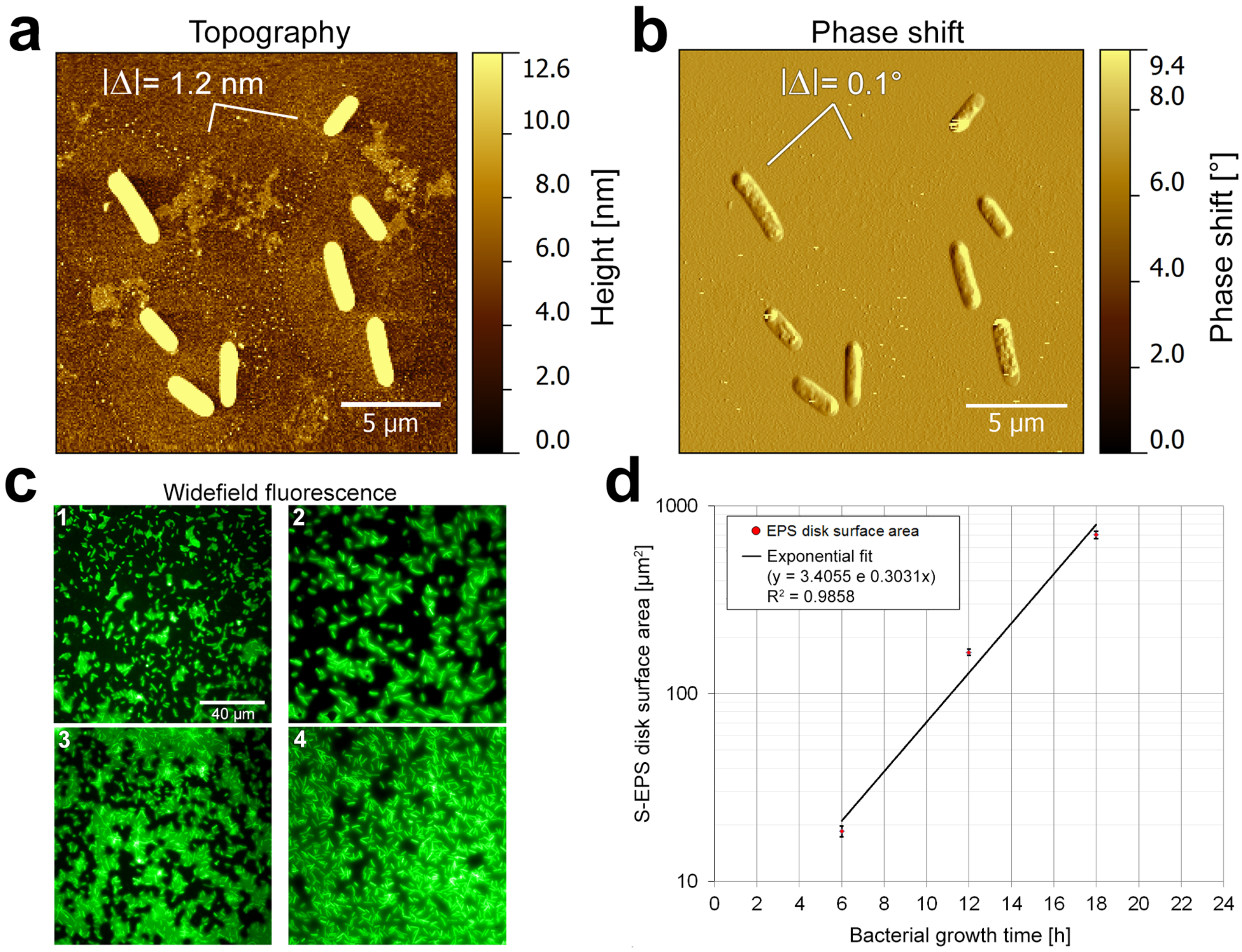

Supplementary Figure S2 | SPM and WFM fluorescence data of deposited S-EPS surrounding surface-adhered bacteria, related to Figure 1. (**a**) Topography data demonstrate height changes (~ 1.2 nm) associated with S-EPS disks around the bacterial cells. (**b**) Phase image of the same sample show only small changes in material viscoelasticity (phase shift of ~ 0.1°) at the regions of S-EPS disks. (**c**) Low-magnification, *ex-vivo* widefield fluorescence images of adhered bacteria demonstrate continuous growth of bacteria-surrounding S-EPS (in sequence 1 to 4). (**d**) Single exponential fitting (black line; fit parameters are indicated within the inset) of the soluble S-EPS disk-covered surface area in correlation to the bacterial growth time (growth times of 6, 12 and 18 hours).

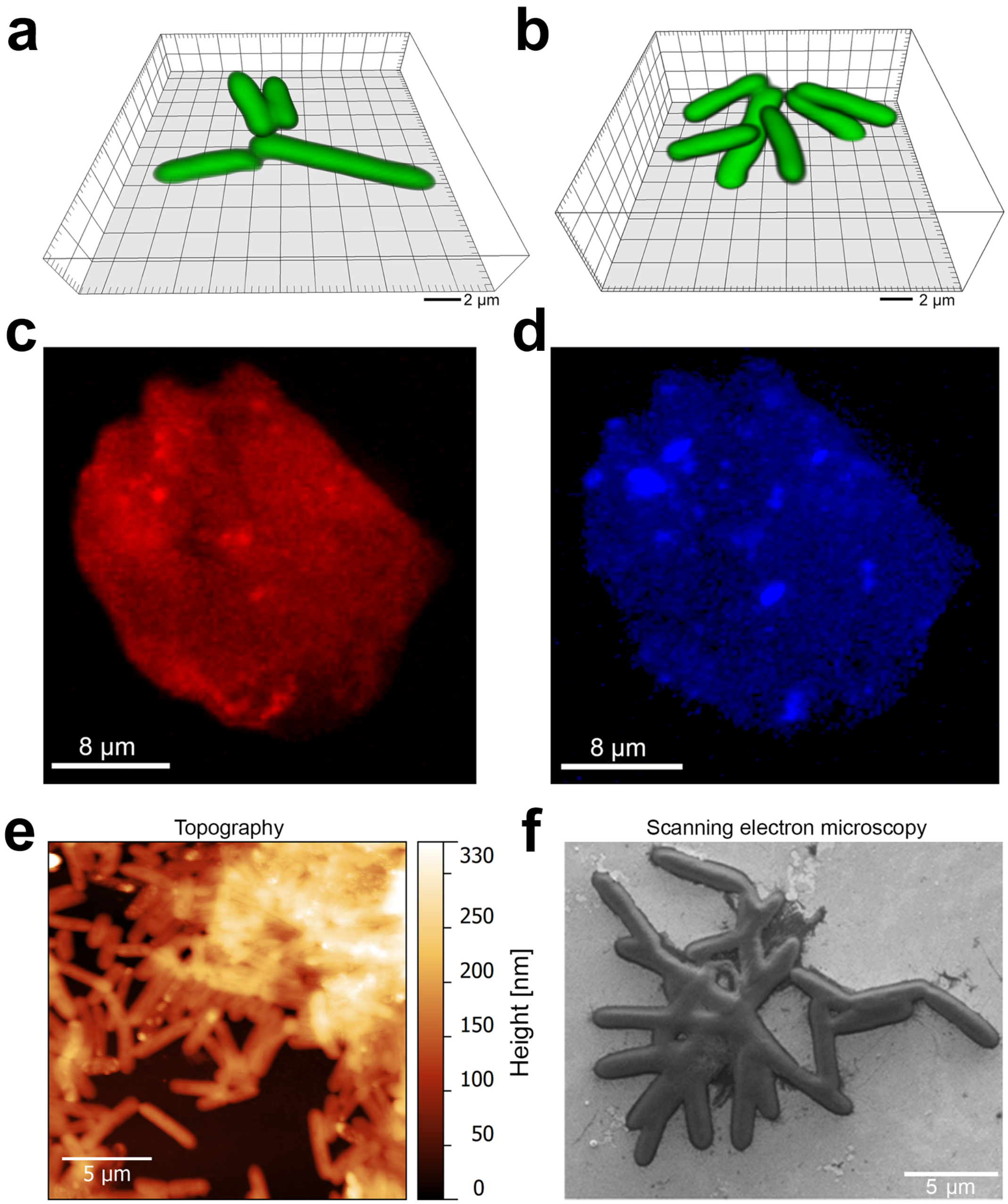

Supplementary Figure S3 | Cell-cell adhesion, formation of LB-EPS covering bacterial clusters and the bacterial deposition of EPS with embedded DNA, related to Figure 2. The *Ex-vivo* SDCLM data (**a**, **b**) of small bacterial agglomerations demonstrate that cell-cell attachment is essentially mediated via the bacterial polar regions. CLSM images of small biofilms show fluorescence emission of Periodic Acid-Schiff-staining (**c**), indicating the existence of neutral polysaccharides within the deposited EPS material. The observed fluorescence of DAPI staining (**d**) identifies also the inclusion of DNA within the biofilm EPS matrix. (**e**) LB-EPS formation on bacterial clusters has been identified via AFM by significant changes in topography. Complementary SEM micrograph of a bacterial aggregate (**f**) reveals the presence of LB-EPS, due to the difficult identification of individual cell borders in the cluster center.

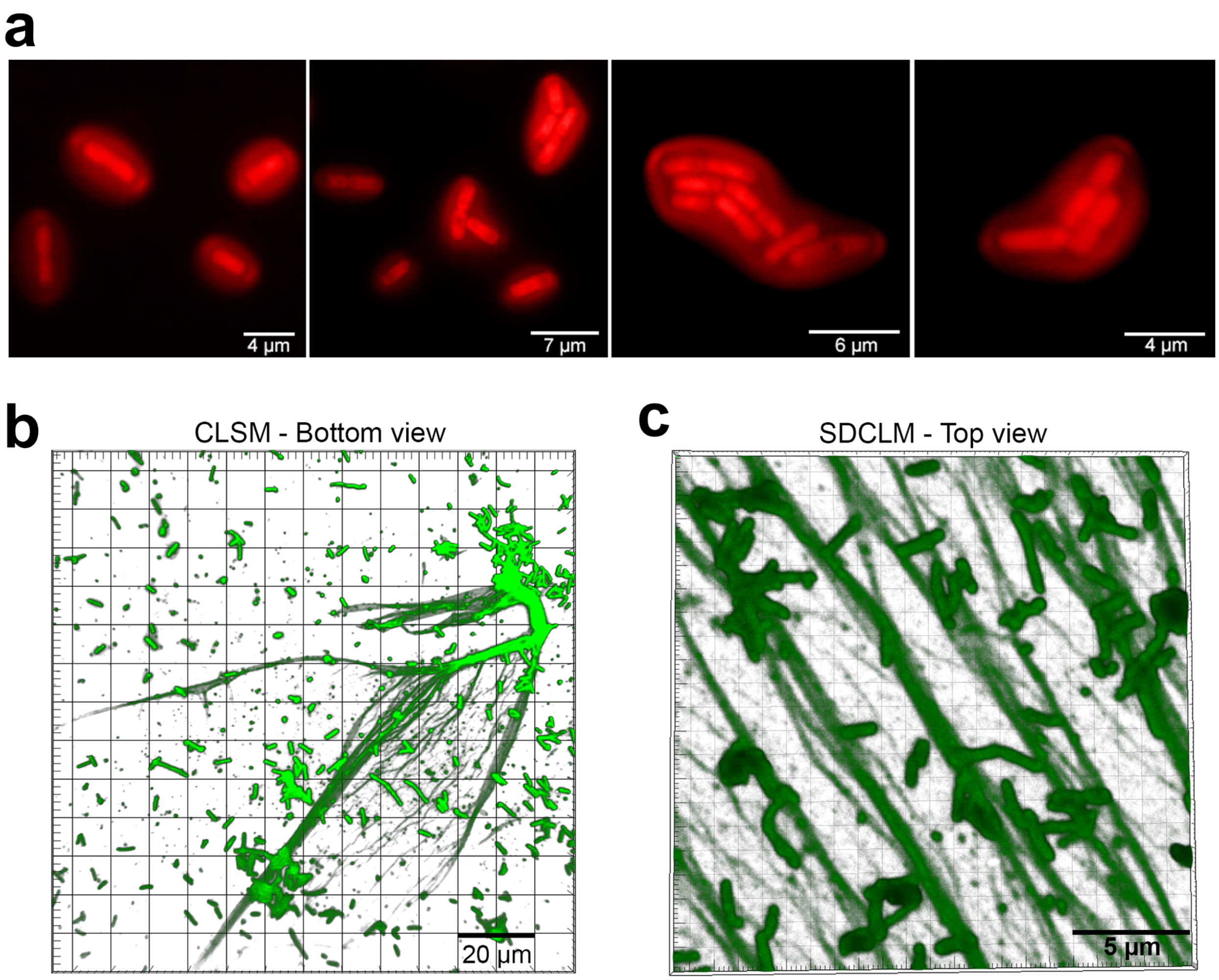

Supplementary Figure S4 | NAG-staining WFM images and e*x-vivo* CLSM and SDCLM images of filamentous EPS structures, related to Figure 4. (**a**) WFM images of WGA-Texas Red staining for small clusters of *X.fastidiosa* cells in a sample grown for 24h; (**b**) CLSM fluorescence image of filamentous EPS structures emerging from highly-dense bacterial clusters. (**c**) SDCLM fluorescence data of higher magnification reveal preferred bacterial adhesion to the small filamentous EPS structural network.

Supplementary Movie S1 | Reversibly surface-attached bacteria, related to Figure 1. Wide-field epifluorescence microscopy recording of GFP-enhanced individual bacteria rotating around its polar region, which is reversibly adhered to the surface.

Supplementary Movie S2 | Cell-cell attachments of bacterial cluster, related to Figure 2. Wide-field epifluorescence recording of a bacterial cluster demonstrating that cell-cell attachment takes place via the polar regions.

Supplementary Movie S3 | Architecture of a young biofilm, related to Figure 3. SDCLM animation of small bacterial clusters after 24 h of growth. Bottom view indicates by their light green color the surface-attached bacteria as cluster anchors.

Supplementary Movie S4 | Mature biofilm architecture, related to Figure 4. SDCLM microscopy animation of a bacterial biofilm after 72 h of growth. The surface anchoring bacteria are visible by their light green color in the bottom view.
